# Estimating Prevalence of Bereavement, Its Contribution to Risk for Binge Drinking, and Other High-Risk Health States in a State Population Survey, 2019 Georgia Behavioral Risk Factor Surveillance Survey

**DOI:** 10.3390/ijerph20105837

**Published:** 2023-05-16

**Authors:** Toni P. Miles, Changle Li, M. Mahmud Khan, Rana Bayakly, Deborah Carr

**Affiliations:** 1 Rosalynn Carter Institute for Caregivers, Atlanta, GA 31709, USA; 2 Department of Health Policy and Management, College of Public Health, University of Georgia, Athens, GA 30602, USA; 3 School of Health Management, Fujian Medical University, Fuzhou 350122, China; 4 Georgia Department of Public Health, Atlanta, GA 30303, USA; 5 Center of Innovation in Social Science, Boston University, Boston, MA 02215, USA

**Keywords:** BRFSS, binge drinking, population survey, bereavement, SDG#3, mental health

## Abstract

Background: Binge drinking is a pattern of alcohol abuse. Its prevalence and associated risk factors are not well documented. Heavy drinking, on the other hand, has a well-documented association with bereavement. This report uses a cross-sectional, population-based survey to estimate prevalence of bingeing and its association with new bereavement. Bingeing is defined as 4 or more drinks (women) or 5 or more drinks (men) in a 2–4-h setting. For the first time in 2019, the Georgia Behavioral Risk Factor Surveillance Survey (BRFSS) included a bereavement item: ‘Have you experienced the death of a family member or close friend in the years 2018 or 2019?’ Methods: Georgia BRFSS is a complex sampling survey administered annually. It is designed to represent the 8.1 million people aged 18 years and older in the U.S. state of Georgia. Alcohol consumption patterns are routinely measured in the common core. In 2019, the state added a new item probing for bereavement in the prior 24 months predating the COVID-19 pandemic. Imputation and weighting techniques were applied to yield the population prevalence rates of new bereavement, bingeing, and their co-occurrence with other high-risk health behaviors and outcomes. Multivariate models, adjusted for age, gender, and race, were used to estimate the risk for other unhealthy behaviors posed by the co-occurrence of bereavement and bingeing. Results: In Georgia, bereavement (45.8%), and alcohol consumption (48.8%) are common. Bereavement and alcohol use co-occurred among 1,796,817 people (45% of all drinkers) with a subset of 608,282 persons reporting bereavement combined with bingeing. The most common types of bereavement were death of a friend/neighbor (30.7%) or three plus deaths (31.8%). Conclusions: While bingeing is a known risk to public health, its co-occurrence with recent bereavement is a new observation. Public health surveillance systems need to monitor this co-occurrence to protect both individual and societal health. In a time of global bereavement, documenting its influence on binge drinking can support the work towards Sustainable Development Goal #3—Good health and Well-Being.

## 1. Background

Bereavement is an established risk factor for morbidity and mortality, yet it has received little attention in prevention research. It is defined as experiencing the death of a significant other, and is different from the concept of grief. Grief is an emotional response to loss such as sorrow, sadness, or anger. Exposure to bereavement within one’s social network is associated with a 2–5-fold increased mortality risk, which can persist for as long as 10 years [1,2]. This exposure also is associated with increased rates of health care consumption [3,4]. Anecdotes describing the behaviors of bereaved people include references to changes in patterns of sleeping and eating, including the harmful subtypes of insomnia and overeating. The emergence of new, detrimental behaviors after exposure to bereavement may be a mechanism driving the downstream increases in health care consumption post-loss. Research describing alcohol-use behaviors shows conflicting patterns of protection and harm. Social drinking protects cognition [5]. Bingeing or heavy drinking increases illness and premature mortality risk [6,7]. This report examines the co-occurrence of bereavement and binge drinking to answer a single question: Is binge drinking significantly more common among people with new bereavement?

Bereavement-related health effects extend beyond biological relatives to encompass coworkers and others with strong social ties, i.e., fictive kin [8]. The strongest evidence for these effects comes from cohort studies. Longitudinal analyses of cohorts show that bereavement is associated with a 2-fold increased risk of death in the 6 years following the event [1]. In these cohorts, rates of detrimental health behaviors—insomnia, smoking, and alcohol use—also were elevated among bereaved persons [9,10,11]. These observations suggest that bereavement presents its pervasive and long-lasting effect in part through behavior change. Can this perspective be extended beyond families to the population level? Evidence suggests that the numbers of people exposed to bereavement is increasing. Population aging and overall population growth have combined with later age at death to increase the annual number of deaths. In the United States, this annual growth in the number of deaths has been observed each year between 1935 and 2010. In 2010, there were almost 2.5 million deaths. By 2018, this number had increased to 2.8 million [12]. Recent work has provided an estimate for numbers of the bereaved linked to a single death from a single cause—COVID. With COVID, an estimated nine people are in the social network of the deceased [13]. Cohort studies, population growth, and cause of death analyses provide indirect evidence for greater numbers of people who have been exposed to bereavement. Is there a parallel trend of emerging, detrimental health behavior alongside these rising numbers of deaths? Answering this question requires a dataset with bereavement exposure combined with health behaviors. Currently, no complex sampling survey connects bereavement and health behavior.

Complex sampling surveys routinely assess health behaviors. The Behavioral Risk Factor Surveillance Survey (BRFSS) has more than 30 years of population-level health behavior assessment. BRFSS measures changes over time to meet current public health concerns. For example, alcohol use only became a regular part of BRFSS in the early 2000s [14]. Bereavement exposure is not routinely assessed in BRFSS. Currently, exposure to bereavement is indirectly inferred from other data sources—population registries or complex sampling surveys of death certificates. [2,3,15]. National mortality registries are a comprehensive listing of deaths. These sources have been used to link bereavement to health care consumption by surviving family members [3]. The National Mortality FollowBack Survey (NMFS) is a complex sampling survey designed to validate death certificates and ascertain events surrounding decedents’ health prior to their deaths. NMFS data are derived from interviews with family members. Despite the sensitive nature of the topic, participation rates range from 90 to 95% in the 3 cycles of NMFS—1966, 1986, and 1993. NMFS does not include health behaviors for the informant. The Health and Retirement Survey (HRS) is a longitudinal cohort study [16]. Health outcomes—not behaviors—are the primary focus of HRS. HRS-based analyses have identified the mediators and moderators of health care consumption after bereavement [1,4,9,10]. The concept of Mastery—global, health, and financial—is a composite attitude index that is linked to the probability of health care encounters such as doctor visits and overnight hospital stays [10]. The timing of exposure to bereavement in HRS is not precisely characterized well enough to evaluate hypotheses regarding behavior change pre- and post-bereavement. HRS captures familial deaths occurring in childhood through the ones occurring between waves of interviews for adults aged 50 years and older. To ascertain changing health behaviors, the timing of exposure and behavior needs temporal standardization. Existing registries or NMFS data also has limited potential for exploring this topic because each is missing informant health behaviors.

Alcohol consumption and abuse are well-studied health behaviors. However, binge drinking and its association with bereavement is a new area of study for population health [11,17]. The pervasiveness of binge drinking creates an ideal starting place for a study of health behavior change as a mechanism for bereavement-related injury. Bingeing is a global issue with rates that vary across nations, ranging from 12.6% (Singapore) to 40.4% (Mexico) [18,19]. Binge drinking is part of a larger spectrum of excess alcohol use. According to the Centers for Disease Control and Prevention, in the U.S., excess alcohol use costs $28 billion in health care, $179 billion in workplace productivity loss, $13 billion in automobile accidents, and $25 billion in criminal justice. These reports indicate that reductions in detrimental consumption could have a large positive effect on a broad spectrum of outcomes—not just health [3,20]. In the United States, annual cross-sectional surveys show increasing rates of bingeing between 2011 and 2017 from 16.7% to 18.0% [21]. Rates also vary by state and region in the U.S., with the highest rates in the Midwest region (20.0%) and in small metropolitan areas (17.7%) [22]. Between 2011 and 2014, the state of Georgia (southeastern region) reported bingeing rates between 13.1% and 16.6%. Traditional studies of bingeing focus on age at first use and its contribution to subsequent heavy drinking [23,24]. While the prevalence of bingeing is well-documented, less is known about the individual, social, and contextual factors that initiate bingeing [25,26]. 

Before the COVID-19 pandemic, the U.S. state of Georgia began field evaluating an item in the BRFSS, estimating the prevalence of new bereavement in the years 2018 and 2019, with the question “Have you experienced the death of a family member or friend in the years 2018 or 2019?” The prevalence of bereavement for that period was 45.8% in a population of 8,164,018 adults aged 18 years and older [27]. Georgia BRFSS contains the necessary elements to study the co-occurrence of binge drinking and bereavement. Its population-level design creates an opportunity to measure the scale of bereavement and behavior change.

## 2. Materials and Methods

### 2.1. Design and Setting of the Study

The 2019 Georgia BRFSS field survey, administered by the Georgia Department of Public Health in the United States, is the data set used for this analysis. The BRFSS is a telephone interview of U.S. residents in all 50 states, and it is a primary source of information on major chronic health conditions, health-related risk behaviors, and the use of preventive services among adults. Alcohol-related items are part of the core set of questions asked by all states. States can also add items of local interest. Georgia added a bereavement module to the 2019 field survey [27]. The core interview takes an average of 17 minutes, while the state-optional items add 5 to 10 minutes. Using list-assisted, random digit dialing, people are randomly selected from the non-institutionalized adult population, aged 18 years and older, of residents in Georgia for interviews. Among the interviews, one is obtained from households drawn from within primary statistical units and includes both landline and cellular phones. The common core contains uniform survey items, asked in all states, on health risk behaviors, chronic diseases, access to health care, and use of preventive services. Due to the differential loss of responses to both individual core and state-added modules, 917 people are missing from the group responding to the bereavement item in 2019 BRFSS.

The binge drinking rates are derived from weighted, imputed BRFSS data for the portion of people reporting any alcohol use (*n* = 6796). Each category of usage is mutually exclusive. For purposes of this presentation, only bingeing and social drinking rates are shown. To compare categories of age, crude rates are calculated using weighted data. These rates illustrate the differences in patterns of drinking between mutually exclusive age categories. The estimated numbers of state residents for each age subgroup are also shown. Crude rates are also used for the multivariate models. To compare gender and race groups, rates were age standardized. Since 1999, the 2000 U.S. standard population has been used by government agencies for calculating age-adjusted rates [21,22]. This approach allows for the comparison of rates across time, without bias, due to population aging. To facilitate comparisons of 2019 BRFSS drinking rates with prior reports, the tables use the 2000 standard population to calculate age-standardized rates. For more details about age-standardized rates see https://seer.cancer.gov/stdpopulations/2000stdpop-use.html (accessed on 9 May 2023).

### 2.2. Measures

In BRFSS, the standard format for health behavior items is structured to capture the 30 day period prior to the day of interview (physical activity, binge drinking, self-rated health, and physical or mental health).

### 2.3. Bereavement

Bereavement was assessed with an optional state-interest item in the 2019 Georgia BRFSS. Bereavement is defined by three items: ‘Have you experienced the death of a family member or close friend in the years 2018 or 2019?’ When the answer was yes, follow-up items included the number of losses and the kinship category of the decedent. The number of losses was coded into four mutually exclusive categories: 0, 1, 2, and 3 or more losses. Relationship to the decedent has three mutually exclusive categories: family member only; friend or neighbor only; both family and non-family member. These items were derived from the HRS [16].

Binge drinking was assessed with the question: ‘Considering all types of alcoholic beverages, how many times, during the past 30 days did you have X (X = 5 for men, X = 4 for women) or more drinks on an occasion?’ We constructed a dichotomous indicator that equals one if a male reported five or more drinks on one occasion or a female reported four or more drinks on one occasion during the past month. The category of social drinking contains people who say ‘yes’ to alcohol use but do not meet either criteria for bingeing or heavy drinking (>7 drinks per week).

### 2.4. Alcohol Screening and Brief Intervention (ASBI)

The Georgia BRFSS included an optional alcohol screening and brief intervention (ASBI) module. Only participants reporting a physician visit in the two years prior to survey were eligible for ASBI. The sample in the ASBI analysis is limited to a subset of 5497 people who also reported a visit with a health care provider, regardless of their drinking habits. The ASBI asks three questions: (1) At that checkup, were you asked in person or on a form if you drink alcohol? (2) Did the health care provider ask you in person or on a form how much you drink? (3) Did the health care provider specifically ask whether you drank (5 for men/4 for women) or more alcoholic drinks on an occasion? Response options for each item: Yes, no, do not know/not sure, or refused.

### 2.5. Sexual Orientation Items

The GA BRFSS also has a test module with items on sexual orientation and gender identity. Sexual orientation is defined by two questions. Which of the following best represents how you think of yourself, and do you consider yourself to be transgender? Response options: gay, straight, or bisexual or something else. Response options for the transgender question are: transgender male to female, transgender female to male, or transgender nonconforming. The Appendix A contains the wording of questions used in the survey.

### 2.6. Statistical Analyses

All statistical analyses were conducted using Stata Version 17 [28]. In the unweighted sample, 4289 respondents had complete information on all 15 items of interest, as shown in Table 1. Chi Square tests were used to detect statistically significant differences. There are several items with no missing responses (gender, age), low missing response rates (education/0.48%, self-rated health/0.33%), and intermediate missing rates (race/2.37%, health behaviors/6–11%). Sexual orientation and gender identity (SOGI) have the highest rates of missing responses (SOGI 25%). When confronted with missing responses across multiple variables, we chose to apply multiple imputation techniques. The applied multiple imputation technique included an assumption of missing at random [29,30]. Multiple imputation allows researchers to use more available data, thus reducing biases due to missing responses [31]. The process began by creating 50 copies of the dataset to reduce the sampling error due to imputations. Next, we used the multiple imputation by chained equations (MICE) approach to impute missing data in multiple variables based on a set of univariate imputation models. These models were conditional models based on the type of variables. For example, the MICE allows for the use of logistic regression models to impute binary variables such as bereavement. Moreover, ordered logistic and multinomial logistic regression models can impute ordered categorical variables, such as educational attainment, and unordered categorical variables, such as race.

Weighted crude and age-standardized prevalence rates were also calculated using the multiple imputation process for sub-groups. Age-standardized rates were calculated using the 2000 U.S. population and formulas. The 2000 standard facilitates comparison with older studies of prevalence. Logistic regression models were adjusted for age and race to generate adjusted odds ratios.

## 3. Results

### 3.1. Bereavement

Analyses included 4289 people with complete information on all variables. Among respondents to the bereavement module, the distribution is as follows: 56.5% females, 91.9% cisgender; 65.7% White, non-Hispanic, and 20.8% Black, non-Hispanic, with a median age of 38 years. Table 1 and Table 2 are focused on bereavement reporting in this survey, and they are organized by Demographics, Social Determinants, and Health Behaviors. The Alcohol Screening and Brief Intervention (ASBI) items were included in a state-optional module focused on health insurance and care utilization.

Table 2 compares estimated bereavement rates and their standard errors. Estimates presented in Table 2 and all subsequent tables are derived from the sample of 4289 participants with complete responses. The projected population of Georgia, aged 18 years and older, is 8,164,018—consistent with the U.S. Census Bureau projections for the state. Planning for resources needed by the population can be estimated using these numbers. The proportion of demographic and geographic subgroups are also included in the table. People reporting the highest rates of bereavement self-identified as Black or African American (57%), people unable to work (52%), or who are unemployed (48%). Statistically significant rates of bereavement are also observed among people living outside of the Atlanta Metropolitan Statistical Area (47%), people who did not complete college or technical school (47%), and females (46%). The final six rows in Table 2 show bereavement rates for high-risk categories of health behaviors—no physical activity, currently smoking, binge drinking, and poor self-rated health. These rates and numbers can be useful for county governments administering services for people with chronic illnesses and disabilities. For each of these high-risk health states, bereavement rates are significantly greater than the overall state rate of 45%. No physical activity is the only exception to this trend. BRFSS also probes physical and mental health. Bereavement rates for people reporting 14 or more days of poor physical health or poor mental health are significantly greater than all others.

### 3.2. Alcohol Consumption

#### Binge Drinking

Table 3 shows the numbers and rates of binge drinking for the 4 million people who reported any alcohol consumption. The first seven rows, labeled Age, present crude rates of consumption for each age category. Age is significantly associated with patterns of consumption. The youngest participants—aged 18 to 20 years—present the highest rate of bingeing (55.6%). Rates are significantly lower in subsequent age groups, down to a low of 14.12% among people aged 65 and older. The legal age for drinking is 21 years in Georgia. Despite that legal barrier, there are 163,860 individuals who drink and are younger than the legal limit. The remaining demographic categories—gender identity, sexual orientation, and self-reported race—show rates of bingeing using age-standardized rates. Males (27.4%) present significantly greater rates than females (20.8%), and rates among LGBTQ persons (25.6%) are significantly greater than among straight persons (24.5%). Among race and ethnicity categories, Black people have significantly lower rates of bingeing than Whites (21.5% versus 25.8%).

### 3.3. Alcohol and Bereavement

Table 4 data present estimates of the co-occurrence of binge drinking (columns) and the intensity of bereavement (rows) for the population of 4 million people who drink. Reports of social or heavy drinking are not shown in the table. People without bereavement show a binge drinking rate of 23.6%. The rates of bingeing among people reporting one death are not significantly different than those without a death (23.8%). People reporting three deaths show significantly higher rates of bingeing (31.8%). Table 4 also shows rates of bingeing within mutually exclusive categories of kinship—family only versus friend or neighbor (fictive kin). Fictive kin deaths are associated with significantly higher rates of bingeing (30.7%) compared to family deaths (19.8%). 

### 3.4. Alcohol, Bereavement, and Health Behavior

#### Bingeing, Bereavement, and Health Behavior

Table 5 is organized so that the reader can compare bereavement rates for high and low-risk states of health behavior among binge drinkers. These rates are age-standardized to remove biases attributable to age. Each health behavior category is shown as a yes or no—currently smoking, fair/poor self-rated health, 14 days of not good mental health, and 14 days of not good physical health. All health behaviors shown in the table share a 30-day reference period. People with combined high-risk health states who also binge drink also have significantly higher rates of bereavement. The bereavement rate is greater in the category of combined smoking and binge drinking (43.0%) versus binge drinking but not smoking (21.5%). Bereavement is more prevalent among those with a combination of bingeing and poor self-rated health (30.1%), combined bingeing and poor mental health (33.7%), or combined bingeing with poor physical health (31.3%). These are cross-sectional associations—not causal. However, the consistent association of bingeing, poor health, and loss suggests a potential mechanism linking health care utilization after bereavement. What do respondents say about being screened by a physician for alcohol use (Any use)? These reports show a paradoxical pattern. Table 5 hints that provider screenings for bereavement may yield insights concerning binge drinking during a health care encounter. The question about any alcohol use includes queries about quantity and, specifically, bingeing. These data were included in Table 5 because other reports show that newly bereaved people are more likely to visit their physician.

If you could ask one question and uncover other health risks, what would it be? Table 6 shows that recent bereavement in the prior 24 months might be that question. In Table 6, each model estimates the adjusted odds ratio (AOR) for a singular high-risk health state—physical inactivity, smoking, poor overall health, poor physical health, and poor mental health. All models are adjusted for age, gender, and race/ethnicity. The row labeled control (Row 1) reminds readers that people who neither binge nor are bereaved are included in the analysis for comparison. People who binge but are not bereaved (Row 2) have a 2.37 times greater rate of smoking and 2.00 times greater rates of poor mental health. In comparison, people who binge and are also bereaved have a greater rate of smoking than bingeing alone—5.14 AOR. The risk for co-occurrence of poor mental health also increases from an AOR of 2.0 to 3.28. The AOR suggests that screening for bereavement could lead to the detection of other high-risk health states.

## 4. Discussion

When queried about the death of a member of their social network in the prior 24 months, people who respond ‘yes’ are significantly more likely to report binge drinking, relative to persons not reporting a bereavement. This observation advances our understanding of the co-occurrence of bereavement and alcohol abuse. This is an advance in the measurement of bereavement for public health purposes. While alcohol abuse rates are an ongoing component of annual surveys, the co-occurrence of alcohol abuse and bereavement was not known. These results advance the search for triggers initiating negative health behavior. The initial rationale for examining bereavement and its negative health effects grew out of a hypothesis that a trend, termed mortality compression, can measurably affect population health. Mortality compression is a shorthand reference to the phenomenon of progressively later ages at death [32]. It is part of the process leading to longer life expectancies, and it is associated with a paradoxical growth in the numbers of people with an increased risk of dying due to advanced age. The potential limitation associated with measuring bereavement in a surveillance survey was a concern about item response rates. A separate analysis examined bereavement reporting items for sampling errors, low precision, non-response, missing data, and biased response rates in small sub-samples. While these issues could limit the use of multivariate modeling required for this report, they were not found to be a significant limitation [27]. Future studies of alcohol abuse, and its association with exposure to bereavement, should begin by replicating these results.

When data from all U.S. states and territories are combined, the rate of binge drinking is more common among higher income people [22]. While we do not dispute the accuracy of these findings, the results from Georgia show a different trend with respect to household income. The association between household income and patterns of consumption is likely a proxy for multiple social determinants related to place of residence. In individual states, governmental levels, such as counties, income, and state alcohol control policy, also influence the accessibility to alcohol and, potentially, binge drinking. The so-called ‘Sin Taxes” are a strategy promoted by public health advocates to control the consumption of products such as alcohol. However, the effectiveness of an alcohol targeted tax is complicated by the variation in median income, rate of alcohol tax per gallon, and sales tax across the states. Analyzing the contribution of taxes to the probability of binge drinking is beyond the scope of our analysis. If this issue were to be pursued in Georgia, it could begin with a median household income of $61,224, an alcohol tax rate per gallon of $3.79, and a general sales tax of 4%. Data combining all U.S states yield a median of $67,521 and an alcohol tax rate of $13.50. There is no national general sales tax. This suggests that binge drinking in Georgia is not limited by low household income because alcohol taxes are low (Tax Foundation, https://taxfoundation.org (accessed on 15 March 2023)).

At the community level, access to alcohol is also a consideration. Not every state has access through multiple venues. For example, in Virginia, liquors of 50% proof or more—such as whisky—can only be purchased in Alcohol Beverage Control (ABC) stores. Georgia does not have ABC stores. Instead, it is a non-quota, non-control, liquor retailer state. All of its 159 counties are defined as ‘wet’, meaning that alcohol is readily available everywhere—restaurants, bars, and any retail outlet. There are 843 liquor stores in Georgia, with an estimated 1 store per 11,767 people. This is not the case in the adjacent state of South Carolina, where the state liquor control board limits access. In Georgia, there is a density gradient of liquor outlets, with a 2 to 1 ratio of stores located north of Atlanta when compared to stores south of Atlanta. In Georgia, 21 is the legal drinking age. BRFSS data consistently shows underage drinking. Therefore, our finding of a high rate of binge drinking in the 18–20 year group is consistent with an environment where access is broad, and costs are low. Income is not a barrier, in these circumstances, despite the inequity gradient in Georgia ranging from $16,632 annual in Zip code 31901 to $148,480 in Zip code 30327.

Social determinants of health (SDOH) are a feature of alcohol abuse requiring further analyses [33]. This is an important limitation of the current analyses. In addition to the density of outlets, taxing policy, and income inequality, SDOH is a framework that could improve our understanding of community access. Future studies conducting targeted analyses of the co-occurrence of alcohol abuse and bereavement should focus on smaller geographic units. The units could be defined by cost factors, outlets where alcohol is sold, and effective screening strategies for the age of purchasers. A model for this analytic approach has been evaluated to evaluate the co-occurrence of housing instability and rates of homelessness [34]. International Classification of Diseases, Tenth Revision, Clinical Modification (ICD10) recently introduced Z codes to encourage the inclusion of SDOH in electronic health records. The Z63 code explicitly captures alcohol, drug addiction, and the co-occurring death of a family member in the household. To effectively implement this future study, use of the SDOH Z codes will need to be improved in electronic records [35].

This report is focused on binge drinking. There are other patterns of alcohol use and abuse, such as social drinking (seven or fewer drinks per week). In the 2019 Georgia BRFSS, 2.5 million people reported a pattern of social drinking. The rate of social drinking is significantly greater, with each successively older age category reaching a maximum of 81.96% among people aged 65 years and older. Women (50.76%) are significantly more likely to define their pattern as social when compared to men (45.82%). Black people (50.8%) have the highest rates of social drinking when compared to Whites (46.8%). In this report, bereavement co-occurring with social drinking is more common among healthy people—better self-rated health (49.9%), people who do not smoke (50.9%), and those with fewer days of poor physical (48.5%) or poor mental health (49.0%). Future studies could be designed to focus on social drinking and bereavement. Although the label social drinking carries less stigma, it is important to keep in mind that alcohol use is not without risk. For adults with comorbid medical conditions and using multiple medications, any alcohol use is associated with fall [6].

The contribution of bereavement to the prevalence of disease and disability is a significant limitation of public health studies. In addition to pandemics, there are other events that increase the rates of bereavement. Examples include mortality due to climate change disasters such as hurricanes, drought, floods, and earthquakes. Global episodes of civil unrest and war also increase rates of bereavement. Future studies are needed to intentionally measure the effects of these events using the framework of bereavement. This report shows the rate of negative health behavior during bereavement is greater than would be expected by chance alone. Future studies also should examine this question prospectively, to ascertain whether the stress of bereavement triggers bingeing, or whether persons who binge maintain social ties with persons who engage in unhealthy behaviors that increase their own mortality risk. In short, prospective or longitudinal data are necessarily to tease out causal ordering.

A rationale for the present study was the nagging question: how does bereavement lead to negative health events? In population-based research, mechanistic studies require greater precision in defining a period for both the exposure (bereavement) and the behavior (alcohol abuse). The BRFSS items reference an intentional period for the exposure—24 months before the interview—and a period for health behaviors 30 days before the interview. To put it succinctly, do negative health behaviors occur in the 30 days before an interview? Is the prevalence rate of these behaviors significantly greater among the bereaved? These analyses suggest that bereavement does increase the likelihood of co-occurring negative health behaviors. This observation is subject to all the limitations discussed in previous paragraphs, but future studies can be designed to address them.

Future studies focused on health behavior after bereavement can serve other purposes. These studies have the potential to unmask other negative health behaviors and influence multiple mechanisms driving morbidity and mortality after bereavement [36,37,38,39]. Alcohol use literature contains paradoxical reports of both protective and detrimental effects. This conflict between protective and detrimental effects complicates the interpretation of the opposing age and gender trends for bingeing observed in this report. The oldest age category (65 years and older) and females have the highest rates of social drinking. These two groups also have the highest rates of new bereavement—65 years and older (50%) and women (46%). Analyses of this paradox are out of the scope of this report. Future studies are needed to disentangle the effect of moderate alcohol use on cognitive function among older adults [5]. This is directly opposite to the literature describing the fall risk associated with alcohol use [6]. Another paradox is the framing of alcohol use as both an exposure and an outcome for individual health. Several reports provide evidence that excess drinking increases the risk of premature mortality [7]. Young adults and males are at the highest risk for this outcome. There is emerging literature providing evidence that bereaved males of all ages are more vulnerable to hazardous drinking, dependence symptoms, and harmful use [11]. Our results suggest that screening for bereavement is likely to identify people with harmful patterns of drinking—an improvement over screening for alcohol use alone.

There is considerable complexity in alcohol use and abuse within individual biology and across cultures. The genetics of alcohol metabolism vary across individuals and populations [36,37,38,39]. This variability influences clinical and public health definitions of abuse and the manifestation of negative effects. There is a long history of cultural norms and expectations for behaviors during the course of bereavement that can initiate or exacerbate the use of alcohol. In western cultures, funerary rites explicitly include alcohol consumption [37]. This expectation is present, to varying degrees, in cultures worldwide [38,39]. These paradoxes require evaluation and refinement to support the development of population-level interventions to diminish bereavement-related alcohol injuries. Gaps remain in our understanding of the association between new bereavement and binge drinking.

While developing this report, we interrogated the data to explore bereavement and social drinking. We did not include the results from those analyses. It is a behavior that is more common than expected, and it is present among people reporting fewer deaths in their social network. Again, this is an observation worth exploring. However, this is a complicated interaction between measured factors, such as the age of the respondent, and factors not available in the dataset, such as whether or not the death was anticipated. We believe that social drinking is an important mechanism underlying the detrimental effects of bereavement. In general, the prevalence of social drinking increases with age in this survey and in prior reports.

What has this report added to our public health perspective on bereavement and binge drinking? Where do gaps remain? These results add to the evidence for poor mental health after the death of friends and family [3,8]. The evidence that bereavement could act to increase the risk of change in negative health behavior is an advance in public health perspectives on the mechanisms driving markers signaling health decline. With this simple idea, future research can measure and target the co-occurrence of bereavement and detrimental alcohol use. This approach can be applied at multiple levels, ranging from a global perspective to something very localized. The World Health Organization, Centers for Disease Control and Prevention, and the Georgia Department of Public Health already have existing strategies for a reduction in bingeing. There are other alcohol control-related policies being evaluated in 194 countries [17]. Sales restrictions were the most common policy implemented across geopolitical blocs. These policies are also consistent with the SAFER initiative—**S**trengthen restriction, **A**dvance drink driving countermeasures, **F**acilitate access to screening, interventions, and treatment, **E**nforce bans or comprehensive restrictions on alcohol sales, and **R**aise prices on alcohol through excise taxes or pricing policies (https://www.who.int/initiative/SAFER (accessed on 15 March 2023)). Target 3.5 of Health-related Sustainable Development Goals (SDG) calls for nations to strengthen the prevention of harmful uses of alcohol. On a county or state level, measuring alcohol outlet density and implementing unit pricing/alcohol taxes is a strategy. This approach demonstrated effectiveness, after the global financial crisis of 2008, in the United Kingdom [40]. Alcohol control policies that increase its price or place limits on amounts for household purchase through a Minimum Unit Pricing policy were effective without being burdensome or resorting to extremes such as prohibition [40]. Responding to widespread bereavement may be a path towards the goal of sustainable prevention of the harmful uses of alcohol [41]. Screening for new bereavement can serve to initiate action at all levels of health care.

## 5. Conclusions

We found that bereavement is associated with a greater likelihood that the respondent also engages in binge drinking. While bingeing is a known risk to health, its co-occurrence with recent bereavement is a new observation. Public health surveillance systems need to monitor this co-occurrence to protect both individual and societal health.

## Figures and Tables

**Table 1:** Variables used in this analysis, complete and missing responses, and variables; 2019 Georgia BRFSS, Unweighted Panel.

Variable	Complete Response, *n*	Complete %	Missing Response, *n*	Missing %
Bereavement item *: Death of family and/or friend, 2018 or 2019.	5206	70.79	2148	29.21
Demographics				
Gender	7354	100.00	0	0
SOGI ^§^	5443	74.01	1911	25.99
Age	7354	100.00	0	0
Race/ethnicity	7180	97.63	174	2.37
Social determinants				
Educational attainment	7319	99.52	35	0.48
Metropolitan Statistical Area, residence	7354	100.00	0	0
Employment status	7202	97.93	152	2.07
Health Behaviors				
Physical activity in the past month?	6780	92.19	574	7.81
Smoking status	6847	93.11	507	6.89
At least one drink of alcohol in the past 30 days?	6796	92.41	558	7.59
Binge drinking	6540	88.93	814	11.07
Self-rated health	7330	99.67	24	0.33
Physical Health not good, days in past month	6802	92.49	552	7.51
Mental Health not good, days in past month	6799	92.45	555	7.55
Alcohol Screening and Brief Intervention (ASBI)				
Asked about any alcohol use	5497	74.75	1857	25.25
Asked how much alcohol	5466	74.33	1888	25.67
Asked about binge drinking	5056	68.75	2298	31.73
Complete information, 15 variables	4289	58.32	3065	41.68

Note: ‘Don’t know,’ ‘Refused,’ and ‘Blank’ equal missing. * New 2019 BRFSS item: ‘*Have you experienced the death of a family member or close friend in the years 2018 or 2019*? SOGI **^§^**: Sexual Orientation and Gender Identity. Module 29, two questions: ‘*Which of the following best represents how you think of yourself? Do you consider yourself to be transgender?* Health behaviors reflect Healthy People 2020 target areas described in https://www.healthypeople.gov/2020/topics-objectives (accessed on 11 April 2021); for all items, see 2019 BRFSS Questionnaire https://www.cdc.gov/brfss/questionnaires/index.htm (accessed on 14 May 2021).

**Table 2:** Percent bereaved within subgroups. The 2019 Georgia BRFSS. Weighted data with Multiple Imputation.

	Estimated Population *n* = 8,164,018	
	Percent	SE
Percent Reporting Bereavement	**45.16**	**1.16**
Demographics		
Males	44.23	1.76
Females	46.03	1.52
SOGI ^§^: CIS Gender	45.46	1.18
SOGI ^§^: All other	41.31	4.97
18–24 years	36.76	5.65
25–34 years	37.42	4.77
35–44 years	42.90	3.33
45–54 years	**47.64**	**2.80**
55–64 years	**47.98**	**2.71**
65 + years	**50.18**	**2.41**
Black or African American only, NH ^€^	**56.07**	**2.42**
White only, NH ^€^	42.17	1.33
All other	33.81	3.42
Metropolitan Statistical County	44.72	1.36
Non-Metropolitan Statistical County	**47.02**	**1.85**
Graduated, College or Technical School	43.38	2.05
Attended College or Technical School	**47.62**	**2.18**
Graduated, High School	45.48	2.20
Did not graduate, High School	42.77	3.04
Employed	44.90	1.61
Unemployed	**48.23**	**5.73**
Retired	45.06	1.91
Unable to work	**52.16**	**3.33**
Homemaker or student	40.35	3.41
High risk states of Health Behaviors in past 30 days		
14 or more days/No physical activity	45.87	2.10
Current smoker/Yes	**53.61**	**3.29**
Binge drinking/Yes	46.12	3.25
SRH ^¥^/Fair/Poor	**50.97**	**2.42**
14 or more days, Physical health not good	**52.56**	**2.69**
14 or more days/Mental health not good	**54.91**	**2.92**

Note: **Bold = *p* > 0.05. Compared to the state rate. Chi χ^2^** new item: ‘Have you experienced the death of a family member or close friend in the years 2018 or 2019? SE = Standard Error, SOGI ^§^, CIS Gender includes ‘I think of myself as straight and not transgender.’ SOGI ^§^, all others includes Gay/Bisexual person/something else and transgender (male to female, female to male, gender nonconforming). NH ^€^ = non-Hispanic. SRH ^¥^ Self-rated health 5 categories: excellent, very good, good, fair, and poor. Health behaviors reflect Healthy People 2020 target areas described in https://www.healthypeople.gov/2020/topics-objectives (accessed on 11 April 2021). For the wording of survey items, see 2019 BRFSS Questionnaire https://www.cdc.gov/brfss/questionnaires/index.htm (accessed on 14 May 2021).

**Table 3:** Crude and age-standardized rates per 100: Binge by Age, Gender Identity, Sexual Orientation, Self-reported Race, and Ethnicity. The 2019 Georgia (GA) BRFSS, Weighted population with Imputation. (Georgia Population, *n* = 8,164,018).

	All Drinkers *n* = 3,988,766	Binge *n* = 1,344,265		
Age (Years)		Rates	CI_Lower_	CI_Upper_
18–20	163,860	**55.61**	**35.65**	**75.66**
21–24	356,944	46.49	34.79	58.58
25–34	878,634	43.69	36.49	50.89
34–44	745,217	36.08	29.25	42.90
45–54	746,216	28.32	22.42	34.22
55–64	579,245	25.75	20.07	31.43
65 +	518,650	14.12	10.34	17.90
Gender Identity/Sexual Orientation, Age-standardized				
Male	2,165,993	**27.40**	**27.34**	**27.46**
Female	1,822,773	20.82	20.77	20.88
Straight	3,673,708	24.48	24.43	24.52
LGBTQ/Other	315,058	25.63	25.45	25.82
Respondent Race, Age Standardized				
Black, NH	1,179,384	21.50	21.43	21.57
White, NH	2,238,967	**25.85**	**25.79**	**25.91**
All other	570,415	**27.72**	**27.58**	**27.85**

**Note**: **Bold numbers, *p* < 0.05**; SOGI and Race reported as standardized rates to the 2000 US Population. Rate per 100 people. CI = 95% Confidence Interval Lower and Upper Limits. Self-identified race. NH = Non-Hispanic Ethnicity. Definitions: Binge drinking: ‘Considering all types of alcoholic beverages, how many times during the past 30 days did you have X drinks*?* (x = 5 for men; x = 4 for women). Social drinking and heavy drinking not shown in the table, *n* = 484,290.

**Table 4:** Age-standardized rates per 100 Binge by response categories to bereavement items—Numbers of deaths reported and Relationship to Decedent. The 2019 Georgia BRFSS, Weighted, Imputed, Total Population aged 18 and older = 8,164,018.

	All Drinkers 3,988,766	Binge 1,344,265	CL_Lower_	CL_Upper_
Number of deaths reported				
None ^¥^	2,191,949	23.60	23.54	23.65
One	837,801	23.83	23.74	23.92
Two	481,214	21.38	21.27	21.50
Three Plus	477,802	**31.80**	**31.66**	**31.94**
Relationship to Decedent				
Family only	852,589	19.81	19.73	19.89
Fictive Kin	372,431	**30.70**	**30.54**	**30.85**

**Note: Bold numbers, *p* < 0.05; ^¥^ Ref = No bereavement;** Rate standardized to the 2000 US Population. Rate per 100 people. CI = 95% Confidence Interval Lower and Upper Limits. Relationship categories are mutually exclusive. Binge drinking: ‘Considering all types of alcoholic beverages, how many times during the past 30 days did you have X drinks?’ (x = 5 for men; x = 4 for women); social drinking and heavy drinking.

**Table 5:** Age-standardized bereavement rates per 100 binge drinkers, by categories of Health-Related Behaviors and Screening by Physicians; 2019 Georgia BRFSS, Weighted, Imputed.

	Georgia Population	Binge Drink	CI Lower	CI Upper
Current smoker?				
Yes	513,774	**43.04**	**42.88**	**43.20**
No	3,474,992	21.52	21.48	21.56
Self-rated health fair or poor				
Yes	594,897	**30.11**	**29.98**	**30.24**
No	3,393,869	23.31	23.27	23.36
Mental Health, not good for 14 or more days in past 30 days				
Yes	629,842	**33.76**	**33.63**	**33.90**
No	3,358,924	22.50	22.46	22.55
Physical Health, not good for 14 or more days in past 30 days				
Yes	374,243	**31.30**	**31.12**	**31.48**
No	3,614,523	23.86	23.81	23.90
Alcohol screening and Brief Intervention (ASBI), Doctor asked				
	Any use?			
Yes	2,672,093	23.69	23.64	23.74
No	1,316,673	**25.72**	**25.64**	**25.80**
	Quantity?			
Yes	2,488,729	22.97	22.92	23.02
No	1,500,037	**26.44**	**26.37**	**26.52**
	Bingeing?			
Yes	1,437,689	24.44	24.37	24.52
No	2,551,077	24.39	24.34	24.45

**Note: Bold numbers, *p* < 0.05;** rates are standardized to the 2000 US Population. Rate per 100. CI = 95% Confidence Interval lower and upper limits. Binge drinking: ‘How many times during the past 30 days did you have X drinks?’ (x = 5 for men; x = 4 for women). Source: https://www.healthypeople.gov/2020/topics-objectives (accessed on 9 May 2023).

**Table 6:** Binge drinking, bereavement, and their combined effects, as well as risky health behavior; 2019 Georgia BRFSS, Weighted with Imputation (*n* = 4,995,641).

	Model 1: Inactivity		Model 2: Current Smoker		Model 3: Poor SRH		Model 4: PH Poor		Model 5: MH, Poor	
Groups	**AOR**	**95% CI**	**AOR**	**95% CI**	**AOR**	**95% CI**	**AOR**	**95% CI**	**AOR**	**95% CI**
Control	Ref		Ref		Ref		Ref		Ref	
Binge, only	0.79	0.54–1.16	**2.37**	**1.47–3.81**	0.81	0.52–1.28	0.79	0.49–1.28	**2.00**	**1.26–3.15**
Binge and Bereaved	1.03	0.69–1.54	**5.14**	**3.39–7.79**	1.00	0.66–1.54	0.93	0.56–1.56	**3.28**	**2.17–4.97**

**Note: Bold, *p* < 0.01, AOR =** adjusted Odds ratio. AOR uses rates standardized to the 2000 US Population. Categories of co-occurrence are adjusted for age, gender, and race. **Control:** 3,676,728 people with no binge drinking and who are not bereaved. **Binge:** Binge drinking, no bereavement. **Binge and Bereaved:** Co-reported binge drinking and bereavement. All health behaviors use the past 30 days as a time reference. Poor uses 14 or more days in the 30 days prior to interview. Health behaviors: https://www.healthypeople.gov/2020/topics-objectives (accessed on 9 May 2023).

## Data Availability

Data are de-identified and publicly available. [RRID:SCR_012974] This data can be found in the Centers for Disease Control and Prevention, https://www.cdc.gov/brfss/data_tools.htm (accessed on 15 March 2023) [RRID: SCR_012976].

## Abbreviations

BRFSS: Behavioral Risk Factor Surveillance Survey; NMFS: National Mortality FollowBack Survey; HRS: Health and Retirement Survey; ASBI: Alcohol Screening and Brief Intervention; SOGI: Sexual orientation and gender identity; MICE: Multiple imputation by chained equations; AO: Adjusted Odds Ratio; SDG: Sustainable Development Goals.

## Appendix A

Gender, Sexual Orientation Questions of the BRFSS, by Year Sex Question (Demographics Section); Sex Question (Screening Section) 2019: Are you male or female?

SOGI Optional Module 2018–2019:

For male respondents

Which of the following best represents how you think of yourself?

1 = Gay
2 = Straight, that is, not gay
3 = Bisexual person
4 = Something else
7 = I do not know the answer
9 = Refused Ask if Sex= 1.

For female respondents

Which of the following best represents how you think of yourself?

1 = Lesbian or Gay
2 = Straight, that is, not gay
3 = Bisexual person
4 = Something else
7 = I do not know the answer
9 = Refused

Do you consider yourself to be transgender?

1 Yes, Transgender, male-to-female
2 Yes, Transgender, female-to-male
3 Yes, Transgender, gender nonconforming
4 No

Source: https://www.cdc.gov/brfss/data_documentation/pdf/BRFSS-SOGI-Stat-Brief-508.pdf (accessed on 24 March 2022).
